# Antibacterial titanium nano-patterned arrays inspired by dragonfly wings

**DOI:** 10.1038/srep16817

**Published:** 2015-11-18

**Authors:** Chris M. Bhadra, Vi Khanh Truong, Vy T. H. Pham, Mohammad Al Kobaisi, Gediminas Seniutinas, James Y. Wang, Saulius Juodkazis, Russell J. Crawford, Elena P. Ivanova

**Affiliations:** 1 School of Science, Faculty of Science, Engineering and Technology, Swinburne University of Technology, PO Box 218, Hawthorn, Victoria, 3122 Australia; 2 School of Engineering, Faculty of Science, Engineering and Technology, Swinburne University of Technology, PO Box 218, Hawthorn, Victoria, 3122 Australia; 3 Center for Nanotechnology, King Abdulaziz University, Jeddah 21589, Saudi Arabia

## Abstract

Titanium and its alloys remain the most popular choice as a medical implant material because of its desirable properties. The successful osseointegration of titanium implants is, however, adversely affected by the presence of bacterial biofilms that can form on the surface, and hence methods for preventing the formation of surface biofilms have been the subject of intensive research over the past few years. In this study, we report the response of bacteria and primary human fibroblasts to the antibacterial nanoarrays fabricated on titanium surfaces using a simple hydrothermal etching process. These fabricated titanium surfaces were shown to possess selective bactericidal activity, eliminating almost 50% of *Pseudomonas aeruginosa* cells and about 20% of the *Staphylococcus aureus* cells coming into contact with the surface. These nano-patterned surfaces were also shown to enhance the aligned attachment behavior and proliferation of primary human fibroblasts over 10 days of growth. These antibacterial surfaces, which are capable of exhibiting differential responses to bacterial and eukaryotic cells, represent surfaces that have excellent prospects for biomedical applications.

Titanium and titanium alloys have emerged as being the most popular choice of implant materials for a range of medical applications, including orthopedic and dental implants^1^,^2^,^3^,^4^, due to their biocompatibility, chemical inertness, osseointegration and excellent resistance to corrosion. Titanium and titanium based alloys can usually withstand corrosive environments due to the presence of a stable TiO_2_ layer that is rapidly formed on the titanium surface when it is exposed to air^3^,^4^. Despite titanium-based implants having shown a good response in *in vitro* environments, their performance in both the short and long term can be adversely affected by the presence of bacterial biofilms that can be responsible for long-lasting infections. A number of biomaterial-centered and prosthetic implant infections have emerged over the past decade, leading to both the implant failure inside the body and the gradual aseptic loosening of the implant from the connecting tissue lining^5^,^6^. On average, it has been estimated that biofilm associated infections affect approximately 10% of patients having undergone medical implant surgery in the United States, ultimately causing nearly 100,000 deaths^7^,^8^.

A number of different approaches for the prevention of bacterial attachment to medical implant surfaces have been developed, one of which is the physical and chemical treatment of commercially pure titanium surfaces^9^,^10^,^11^,^12^,^13^. Of the chemical methods for fabricating titanium-containing nano-patterned surfaces, hydrothermal treatment^14^,^15^ represents an environmentally friendly technique. This technique is usually carried out under controlled temperature and pressure conditions in an autoclave such that the operating temperature is maintained above that of the boiling point of water to self-generate a saturated vapor pressure^16^. This chemical treatment is followed by heat treatment, which allows the morphology of the passivated layer of titanium oxide to be accurately controlled, producing a uniform surface that contains nano-wires^17^,^18^. The additional benefits associated with this fabrication technique are that the fabrication involves a single procedural stage, with low hydrothermal temperatures being required to enable the complete transformation of the initial titanium surface into one that contains nano-patterned, hierarchical structures^19^.

A recent study reported that titanium surfaces had been modified to possess nano-patterns that had been inspired by the surface topography of cicada wings^20^. This study aimed to impart a different nano-pattern onto the surface of titanium by modifying the parameters used in the hydrothermal treatment process. Although much emphasis has been placed on developing surfaces that have the ability to selectively prevent the attachment of bacteria, exhibit bactericidal activity or allow the proliferation of osteoblast-like cell lines, very little data has been reported in the literature regarding the role that surface properties such as surface topography, morphology or wettability play in the cell attachment process. It has been reported that titanium coupons soaked in alkaline NaOH and KOH solutions were able to impart nanostructured titanate layers on the top surface of the titanium^21^,^22^,^23^. These studies, however, were not exhaustive in that the antibacterial activity of the resulting surfaces was not extensively investigated.

Biomimetics is a technical discipline that endeavors to fabricate artificial structures inspired by those found in nature^24^. Naturally occurring building strategies generate a remarkable compilation of technical equilibrium that is shared amongst many biological materials. These principles of surface growth are rarely found on the surfaces of conventional metals but have the potential to provide desirable properties when used as templates for the natural materials on which they are based^25^,^26^. A great deal of inspiration has resulted from studying the self-cleaning and antibacterial dragonfly wing surfaces^27^,^28^,^29^.

In the current study, titanium surfaces were produced that mimicked the surface architecture of dragonfly wings, with the assumption being made that this surface architecture would possess the same antibacterial properties exhibited by the dragonfly wings. The nano-patterned surface arrays were fabricated via a facile one-step hydrothermal etching process. The resulting surfaces were comprehensively characterized. The results reported here provide further evidence that titanium surface nano-features can be engineered with a view to controlling the extent to which bacterial attachment takes place onto such surfaces. Furthermore, it has been shown that these titanium surfaces have the ability to influence the processes of bacterial attachment and promote the robust, ordered and enhanced attachment of primary human fibroblasts over 10 days of growth, as shown here for the first time.

## Results and Discussion

### Characterization of titanium surfaces

**Figure 1:**
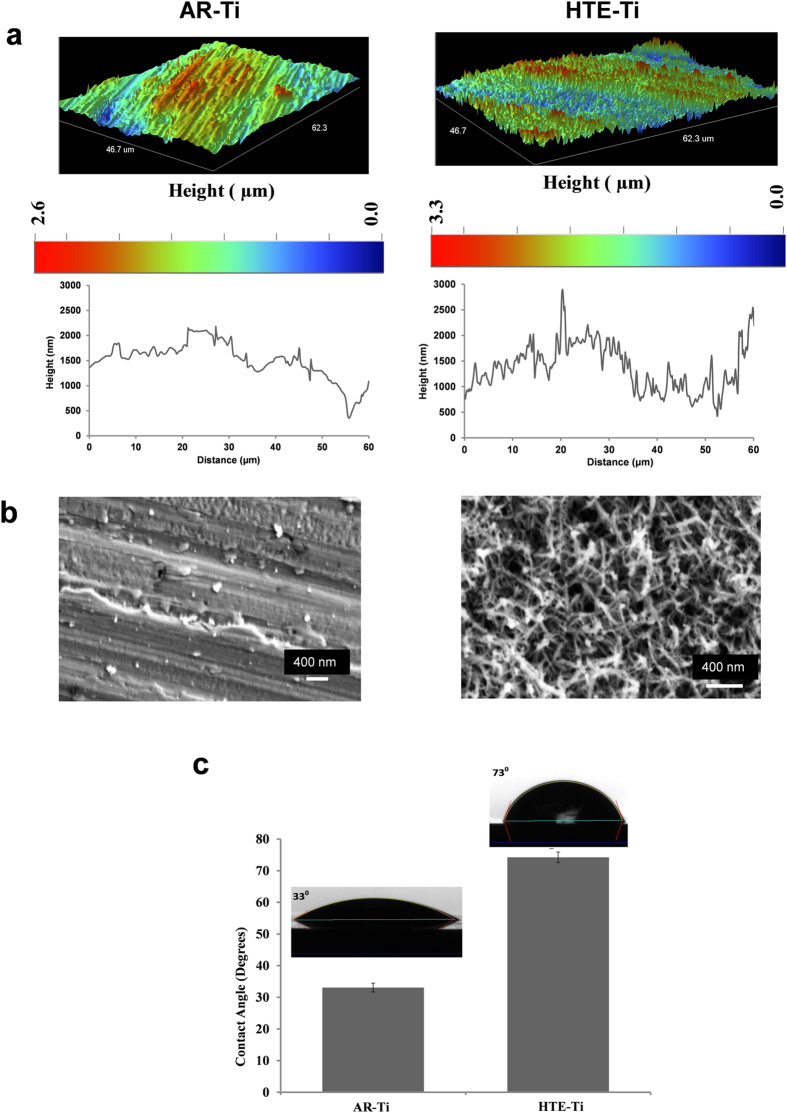
Typical surface characterization images of the AR-Ti and HTE-Ti surfaces. (**a**) High resolution optical profilometry images of the AR-Ti and HTE-Ti surfaces over a scanning area of 46.7 μm × 62.3 μm along with height profiles, indicating the presence of sharp nanowires on the surface of the HTE-Ti. (**b**) Representative SEM images of the AR-Ti and HTE-Ti samples (Scale bar 400 nm). (**c**) Wettability of AR-Ti and HTE-Ti surfaces in terms of their water contact angles.

**Figure 2:**
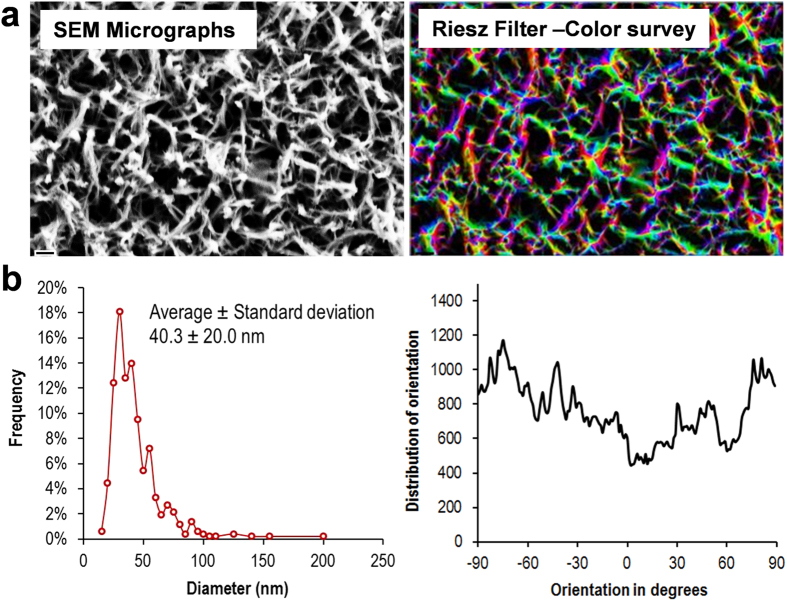
Regularity of nanopatterned arrays on the HTE-Ti surface as analysed using ImageJ software. (**a**) SEM micrographs were transformed and filtered to determine the size and angle orientation of the nano-wire arrays. (**b**) Size distribution (left) and orientation angle (right) of the nano-wire arrays.

Micro-to nano-scale patterning of metallic substrates has been shown to be an effective way by which cell-substrate interactions can be controlled for both prokaryotic and eukaryotic cells^30^. The data pertaining to the topographic and physicochemical characterization of the titanium surfaces, both as-received titanium (AR-Ti) and titanium after being subjected to hydrothermal etching (HTE-Ti), are presented in Fig. 1. An optical profilometer was used to provide an overview of the height of the surfaces as a function of distance over large scanning areas (Fig. 1a). Scanning areas of 46.7 μm × 62.3 μm were visualized for each titanium sample, which was also visualized in three-dimensions. The SEM micrographs revealed that the surface topography was not altered by the hydrothermal etching on the micron scale, however nano-wire arrays were observed to form on the nano-sized features of the AR-Ti (Fig. 1b). The statistical topography and roughness distribution of the AR-Ti and HTE-Ti are presented in Supplementary Table S2. A comparative roughness analysis was performed using the two conventional surface roughness parameters, the average roughness (*S*_a_) and the root mean square roughness (*S*_q_). These parameters showed that the surface of the HTE-Ti (*S*_a_ 401.4 nm) was greater in nano-roughness than the AR-Ti (*S*_a_ 356.9 nm) due to the presence of the nanowire arrays. These nano-wire arrays were found to orient themselves predominantly in a perpendicular configuration to each other as shown in Fig. 2. The average size of the nanowires was estimated to be approximately 40.2 nm. A wettability analysis of the AR-Ti and HTE-Ti surfaces revealed that the HTE-Ti surface was more hydrophobic, exhibiting a water contact angle (θ_W_) of 73° compared to that of the AR-Ti, (θ_W_ = 33°). Surface wettability is an important parameter that can be used to assess the possibility that a surface could exhibit antibacterial behaviour, since many superhydrophobic surfaces have been shown to do so. Surface wettability is usually assessed by measuring the water contact angle, defined by the Young equation, formed at the three phase line of contact when a water droplet is placed in contact with a solid surface. The HTE-Ti surface is slightly more hydrophobic than that of the AR-Ti surface, with the small increase being attributable to the presence of the surface nano-wire arrays formed on the surface, which can entrap air and therefore increase the hydrophobicity of the surface^31^,^32^.

**Figure 3:**
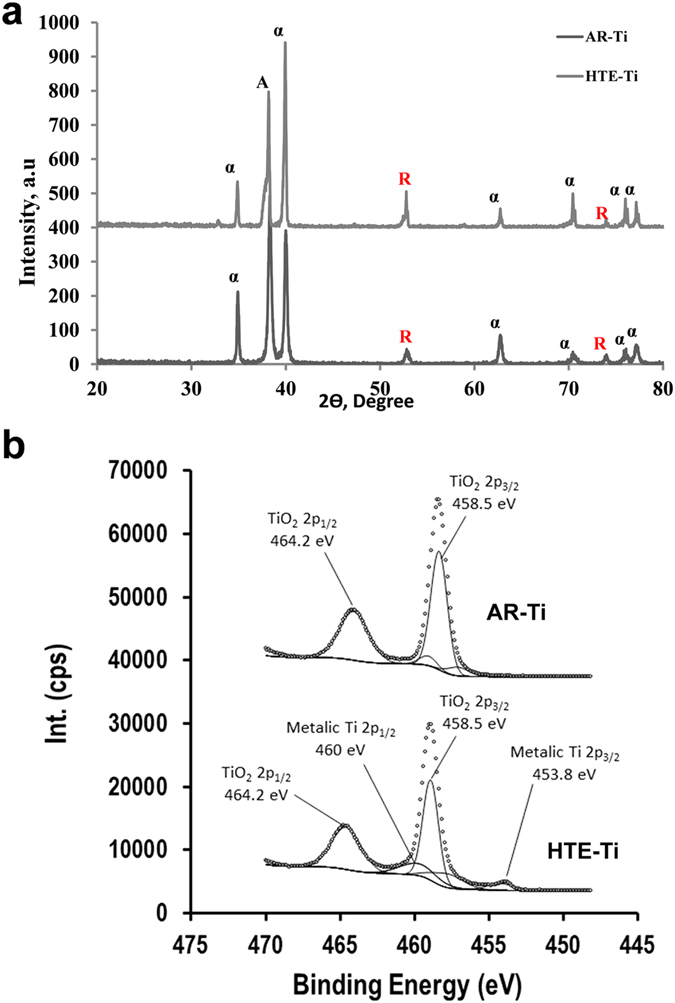
The chemical elemental analysis of the AR-Ti and HTE-Ti substrates. (**a**) X-ray diffraction showing the crystallinity of titanium and titanium dioxide on the AR-Ti and HTE-Ti (alpha (α) phase of titanium, anatase (A) and the rutile (R) phases of titanium dioxide). (**b**) High-resolution XPS spectra of titanium revealing the oxidation states of titanium present on the AR-Ti and HTE-Ti surfaces.

The chemical characteristics of the AR-Ti and HTE-Ti are shown in Fig. 3 and Supplementary Table S1. X-ray diffractograms demonstrated that no clear difference existed between the crystalline phases of the AR-Ti and HTE-Ti, however an enhanced formation of crystalline titanium dioxide on the HTE-Ti was noted, with an increased ratio being observed between the 2*θ* peaks of the anatase phase of titanium dioxide (40°) and the alpha phase of titanium (38°) (Fig. 3a). High resolution XPS analysis indicated the presence of high levels of elemental potassium on the HTE-Ti surface, which likely resulted from the KOH solution etching process. The high-resolution 2p Ti spectra confirmed the formation of titanium dioxide nanowires on the substrate surface from the hydrothermal etching process (Fig. 3b). No significant difference was observed between the chemical properties of the AR-Ti and HTE-Ti surfaces. An advantage of using a hydrothermal etching technique is that the surface morphology can be readily controlled by adjusting the parameters of the etching process, which can be performed without significantly changing the surface chemistry^14^,^33^.

### Selective bactericidal efficiency of titanium surfaces containing nanowires

**Figure 4:**
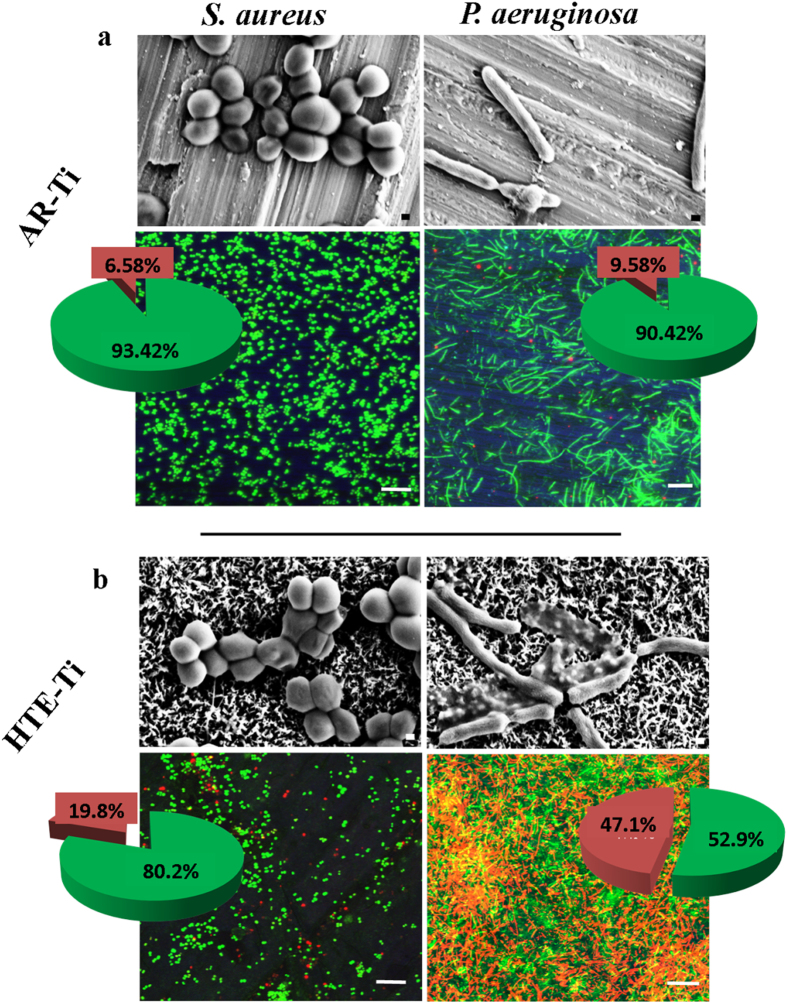
Representative *S. aureus* (left) and *P.* *aeruginosa* (right) attachment patterns on the AR-Ti (top) and HTE-Ti (bottom) surfaces after an 18 h incubation period. SEM images represent an overview of the attachment pattern (Scale Bar: 200 nm). CSLM images reveal the viable (stained green with SYTO 9) and the non-viable cells (stained red with Propidium Iodide) (Scale Bar: 10 μm). The antibacterial activity of both substrates has been shown for both bacteria in the individual pie charts.

The bacterial attachment patterns of two common human pathogens, *Staphylococcus aureus* and *Pseudomonas aeruginosa*, are presented in Fig. 4. Representative SEM images have been used to illustrate the bacterial attachment patterns onto the AR-Ti and HTE-Ti surfaces. The number of viable and non-viable bacterial cells on the substrates was determined using confocal microscopy. Viable cells were stained green with SYTO 9^®^ and non-viable cells were stained red with propidium iodide (Fig. 4). These images revealed that bacterial attachment occurred to a greater extent on the AR-Ti (Supplementary Table S3), with 6.3 ± 2.1 × 10^4^ and 5.2 ± 0.9 × 10^4^ *P. aeruginosa* cells and 42.4 ± 4.9 × 10^4^ and 37.3 ± 3.8 × 10^4^ *S. aureus* cells attaching to the AR-Ti and HTE-Ti per mm^2^, respectively. The total number of cells attaching to the Ti substrates is consistent with that reported previously^34^.

The confocal images also demonstrated that 93.4% of the *S. aureus* and 90.4% of the *P. aeruginosa* cells remained viable after attachment to the AR-Ti surfaces, displaying typical attachment behavior^4^,^35^,^36^. By contrast, 80.2% of the *S. aureus* and 52.9% of the *P. aeruginosa* cells were found to be viable after attachment to the HTE-Ti surfaces (Supplementary Table S4). As can be seen in Fig. 4a,b, a number of the *S. aureus* and a greater number of *P. aeruginosa* cells appeared to be damaged by the action of the nano-wires present on the surface.

**Figure 5:**
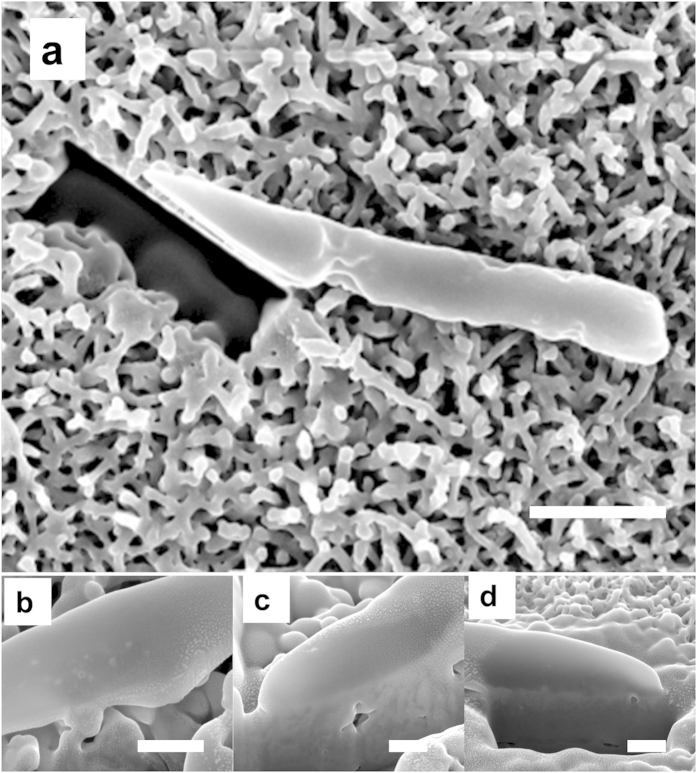
Interaction between the membrane of the *P.* *aeruginosa* cells and the nanowires present on the surface of the HTE-Ti as visualised by FIB-SEM. (**a**) Top view of a *P. aeruginosa* cell on the HTE-Ti surface (scale bar 1 μm). (**b**–**d**) Engulfment of *P. aeruginosa* cell membrane into the nanowires on the HTE-Ti surface (scale bar 200 nm).

The nano-wire arrays present on the HTE-Ti rendered the HTE-Ti as a moderately effective bactericidal surface, with a greater bactericidal activity against *P. aeruginosa* bacteria. This greater activity could be attributable to *P. aeruginosa*, being a Gram negative bacterium, having cell walls that are more susceptible to morphological deformation than those of the Gram positive *S. aureus* cells^37^. This is due to the fact that *P. aeruginosa* bacteria contain a thin peptidoglycan layer in their cell wall, whereas the cell walls of the Gram-positive *S. aureus* bacteria are comprised of many interconnecting layers of peptidoglycan, and hence the cell wall thickness is much greater than that of Gram-negative bacteria^38^. This was confirmed using focused ion beam scanning electron (FIB-SEM) microscopy (Fig. 5), which highlights the ability of the *P. aeruginosa* cell walls to be engulfed by the action of the surface nanowires. This membrane deformation could arise due to the high surface free energy and the hydrophilicity of the nano-wire arrays on the HTE-Ti, where the membrane deformation occurs to balance the high amount of energy gained through the initial attachment of the bacteria onto the HTE-Ti surface. The membrane deformation that occurs leads to the eventual damaged cellular morphology that is observed on the surface^39^.

The results of this study demonstrated that the chemical modification of the surface of titanium can reduce the propensity for some bacteria to attach to the surface. The bacterial attachment patterns of two pathogenic bacteria, *S. aureus* and *P. aeruginosa*, highlighted that not only the attachment on the HTE-Ti occurred to a less extent for both bacteria; the nano-wire arrays present on the HTE-Ti made the surface moderately bactericidal, with a greater bactericidal being demonstrated activity against the *P. aeruginosa* cells.

### The proliferation of eukaryotic cells on titanium surfaces

**Figure 6:**
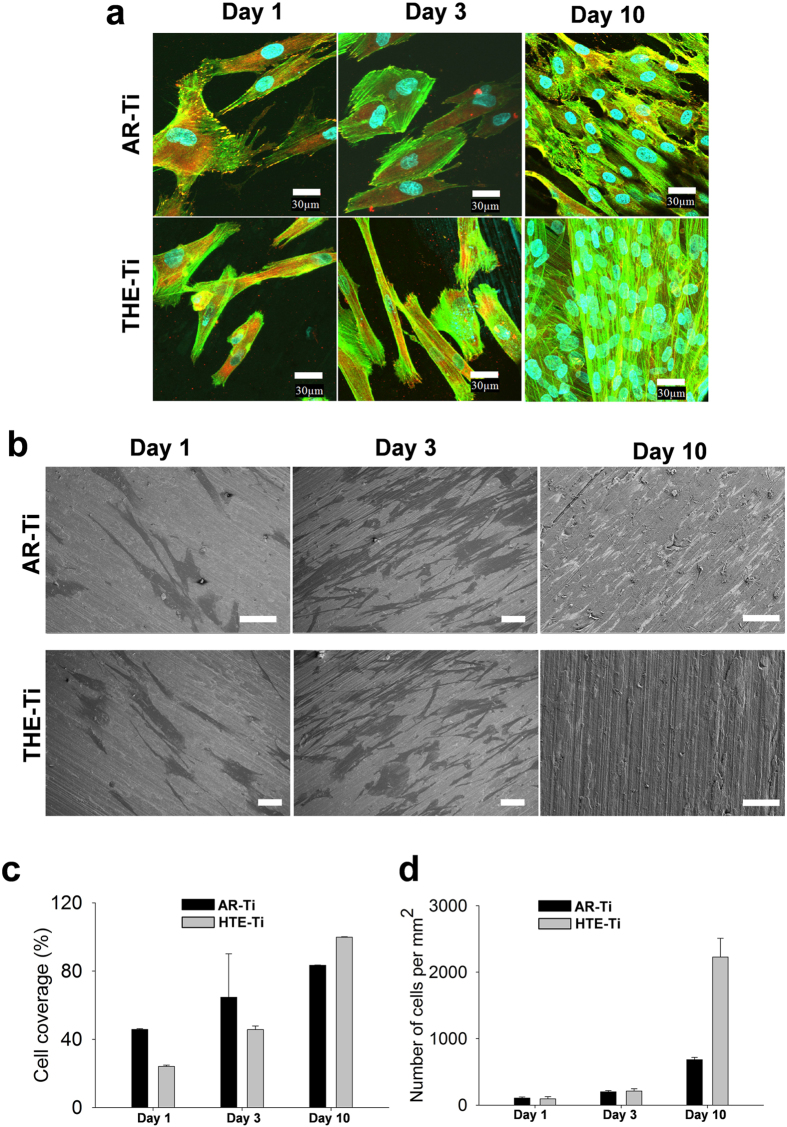
Adhesion and proliferation of human primary fibroblasts (pHF) on the AR-Ti and HTE-Ti surfaces after incubation periods of 1, 3 and 10 days as shown by representative (**a**) CLSM and (**b**) SEM images. The cell coverage (%) and the number of cells attaching to the respective surfaces are given in (**c**) and (**d**).

To enable an *in vitro* analysis of the processes occurring when human cells interact with the surface of a biomedical implant, an in-depth analysis of the mechanisms of cell proliferation and cell morphology was needed in order to understand the various relevant biological processes taking place. Primary human fibroblasts are cell lines that require a suitable substrate for initial anchorage, which is then followed by cell proliferation. If these cell lines do not identify a suitable anchor, their cell division processes become arrested^40^. Human primary fibroblasts (pHF) were incubated in the presence of the AR-Ti and HTE-Ti substrates for 1, 3 and 10 days, the adhesion and proliferation results of which are given in Fig. 6. The number of cells that had attached to the substrate surfaces after an incubation period of 10 days was visualized using scanning electron microscopy and confocal scanning microscopy and compared for the respective substrates under investigation. The pHF cells appeared to successfully adhere to the substrate surfaces after Day 1 and continued to proliferate over the 3 and 10 day incubation periods on both surfaces, with higher rate of proliferation on the surface of the HTE-Ti samples (Fig. 6a,b,d). After three days of incubation, it was also observed that the cells were beginning to align with the nano-wires present on the HTE-Ti surfaces, whereas no such alignment was observed on the AR-Ti surfaces^41^.

**Figure 7:**
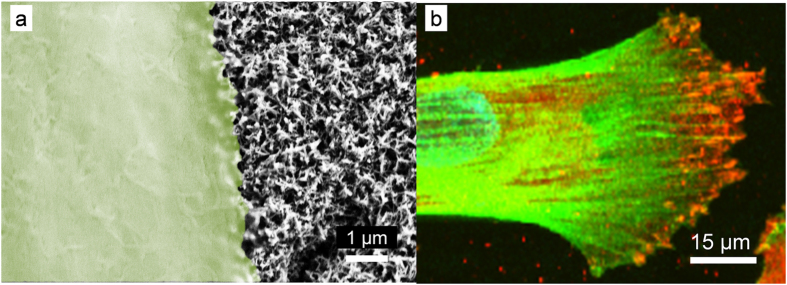
Interaction between primary human fibroblast cells and the nanowires present on the surface of HTE-Ti. (**a**) High-resolution SEM of the surface, highlighting the anchorage points of the membrane onto the surface nanowires, (**b**) CLSM images showing the spreading of primary human fibroblast cells over the surface, with the anchorage points of vinculin (stained with red) (green indicating actin, blue indicating nucleus).

After 10 days of incubation, the pHF cells formed a 90% confluent monolayer on the AR-Ti surfaces, however multiple layers of cells were observed using both SEM and CLSM on the HTE-Ti surfaces. While the pHF cells on the AR-Ti surfaces were found to be evenly distributed and elongated over the 10-day incubation period, the cells were found to exhibit an extended morphology on the HTE-Ti surfaces. This different attachment morphology could be attributed to the nano-wire aligned attachment behavior of the pHF cells on the HTE-Ti surfaces. The nano-wire arrays present on the HTE-Ti surfaces tended to create an elastic force on the eukaryotic cells, causing the cells to shrink and elongate. In contrast, these cells retained their usual rounded morphology on the AR-Ti surfaces. These differences in morphology are consistent with those presented in previously reported data^20^. The finger-like filament extensions visible for the pHF cells attached to the HTE-Ti substrate surfaces, resulting in greater cell coverage (Fig. 6c). The increase in cell attachment to the HTE-Ti surface can be attributed to the ability of the nano-wire arrays on the HTE-Ti to serve as focal adhesion points, which further act as directional growth cues for the fibroblast cells. This elongation of the filaments has also been observed in previous studies, suggesting that the fibroblast cell lines generate extended filopodia when attaching to structured surfaces in order to create a greater number of anchorage points^42^,^43^,^44^,^45^,^46^. This nano-patterned surface texture positively influences the growth and proliferation of cells, which leads to the observed growth contact guidance. Contact guidance influences the proliferation of cells by dictating the orientation of growth along the nano-wires on the surface. After a 10 day incubation period, it was observed that there was no significant difference in the overall cell coverage area on the AR-Ti and HTE-Ti surfaces. There was, however, a significant increase in the number of fibroblast cells that attached onto the HTE-Ti surfaces compared to the AR-Ti surfaces (Fig. 6c,d). Higher cell densities play a critical role in contributing to the stronger adhesion of tissue to the base substratum^47^. The results obtained in this study suggest that the nano-wire containing surface structures of the HTE-Ti surfaces, both on the micro- and nano-scale, could significantly affect the extent of cellular adhesion and proliferation that would take place on such structured surfaces^48^. The focal contact points of the pHF cells and the HTE-Ti surface were detected using high-resolution SEM and immunocytochemistry, the results of which are shown in Fig. 7. The edges of the pHF cells appeared to stretch and anchor to the nano-wire structures on the HTE-Ti surface by extending their cytoskeletal membranes. The SEM image given in Fig. 7a highlighted the local contact area between the cell edges and the surface nano-wires, with some of the nano-wire arrays being visible beneath the edges of the cell. In the confocal micrograph shown in Fig. 7b, these focal adhesion points were able to be detected by the application of anti-vinculin, an antibody that is used for the detection of viniculin. Vinculin is a cytoskeletal protein believed to be one of several interacting proteins that are involved in anchoring F-actin, a cellular protein that forms microfilaments, to the membrane^49^. The adhesion plaque of fibroblasts on any surface is formed by proteins such as vinculin, talin, alpha-actinin and paxillin. Actin filaments or intermediate filaments are conjugated by these proteins to trans-membrane integrin receptors and the extracellular matrix (ECM). Thus, it can be said that these focal contacts are stable points of connection between intra- and extracellular fiber systems. Substratum-sensitive cells such as the fibroblast cell lines endeavor not only to attach, but also to conform to the surface topography^50^. Therefore, it shows that the nano-wired titanium surfaces serve as successful anchorage points for the proper adherence of the eukaryotic cell lines. It was shown in a previous study that nano-fibres less than 100 nm in height are better able to provide guided directionality to the proliferation of the fibroblasts^44^. Conflicting results have also been reported, where fibroblasts have undergone decreased tendency to grow onto modified titanium surfaces^51^,^52^. In these studies, the methods (such as plasma polymerization) used for modifying the titanium substrate surfaces were markedly different than that used in this study. In another study, the plasma polymerization process resulted in a change to the surface chemistry of the titanium^53^.

## Conclusion

In summary, this work describes the fabrication of titanium surfaces that possessed hierarchically ordered titanium nano-patterned arrays with selective bactericidal activity and osseointegration functionality. These surfaces were able to be fabricated by a straightforward and reliable chemical hydrothermal treatment followed by a high temperature treatment. The nano-wire arrays formed on the titanium substrates were found to be strikingly similar to the natural bactericidal nano-patterns found on dragonfly wings^41^. The results generated in this study suggest that an optimal surface modification technique to achieve such a structure would be one that not only increases the ability of the substrate to allow the adherence of human cells, but also increases the bactericidal properties of the surface. While the surfaces reported in this study have exhibited modest antibacterial behavior towards *P. aeruginosa* and *S. aureus*, further modification of the reaction parameters (such as the heat treatment temperature and the concentration of the working solution) would be required for the production of more effective bactericidal surfaces.

## Methods

### Hydrothermally etched titanium nanowire surfaces

Commercially pure ASTM Grade-2 titanium, in billets 10 mm in diameter and 35 mm in length, with an average grain size of 4.5 μm were used as samples in this study. Small disc-shaped specimens were prepared from the as-received titanium rods and were subjected to further hydrothermal treatment to form nano-wire arrays on their top surfaces. The titanium control samples were prepared by progressively grinding their surface using silicon carbide grinding papers. This was to ensure a substrate could be obtained that contained a regular plane surface with slight shallow scratches, free of deformation pits. Hydrothermal treatment of the as-received titanium billets was performed by fully immersing the billets in 10 M KOH solution in a glass container and subjecting them to a high temperature and pressure treatments in a steel autoclave. The temperature and the pressure were 121 °C and 10–15 psi, respectively. The liquid hydrothermal process was performed for a one hour period. The billets were subjected to an additional heat-treatment procedure in a hot air furnace at 400 °C for 3 h. As a final cleaning step for the AR-Ti and HTE-Ti samples, the substrates were rinsed and ultrasonically cleaned, firstly in acetone, ethanol and finally in MilliQ H_2_O (with a resistivity of 18.2 MΩ cm^–1^) to remove any trace impurities. The mechanism by which the nano-wire pattern is formed on the surface is described in the Supplementary Information S2.

### Surface topography

The surface topography of the substrates was analyzed using the Wyko NT1100 (Veeco Instruments, Bruker) optical profiling system in the white light vertical scanning interferometry mode using a 20× objective lens. Three samples of each surface type were briefly scanned to evaluate the overall homogeneity of the surface and then the topographical profiles were studied in detail at five different locations. To investigate the regularity of the nano-wire arrays on the HET-Ti samples, SEM images were obtained using the ImageJ^®^ 1.48 package with DiameterJ and OrientationJ plugins. A more detailed description is included in the Supplementary Information S1.

### Surface chemistry

X-ray Photoelectron Spectrometry (XPS) was performed using an Axis Ultra spectrometer (Kratos Analytical Ltd., UK), equipped with a monochromatic X-ray source (Al Kα, hν = 1486.6 eV) operating at 150 W. The spectrometer energy scale was calibrated using the Au 4f_7/2_ photoelectron peak at binding energy EB = 83.98 eV. During the analysis, the samples were flooded with low-energy electrons to counteract any surface charging that may take place. The hydrocarbon component of the C 1s peak (binding energy 285.0 eV) was used as a reference for charge correction. Photoelectrons emitted at 90° to the surface from an area of 700 μm × 300 μm were analyzed at 160 eV for survey spectra and then at 20 eV for region spectra. Survey spectra were recorded at 1 eV/step, while the region spectra were taken at 0.1 eV/step. The relative atomic concentration of elements detected by XPS was quantified on the basis of the peak area in the survey spectra with the sensitivity factors for the Kratos instrument being used. Peaks in the high-resolution regions of spectra were fitted with synthetic Gaussian-Lorentzian components after the removal of a linear background (using the Kratos Vision II software).

### Wettability

Surface wettability measurements were also conducted via the measurement of water contact angle. The sessile drop method was employed to measure the static contact angles of MilliQ H_2_O (with a resistivity of 18.2 MΩ cm^–1^), with contact angle measurements being performed using an FTA1000 (First Ten Ångstroms Inc.) instrument. An average contact angle, averaged from at least five measurements, was determined for each substrate. Each measurement of a particular contact angle was recorded in 50 images taken within 2 seconds with a Pelco Model PCHM 575-4 camera and the contact angle was determined from the images analyzed by the FTA Windows Mode 4 software.

### Microorganisms, culture conditions, and sample preparation

Because of their clinical significance, *S. aureus* CIP 65.8^T^ and *P. aeruginosa* ATCC 9027 were selected for this study. Samples were obtained from the American Type Culture Collection (ATCC, USA) and Culture Collection of the Institut Pasteur (CIP, France). Bacterial stocks were prepared in 20% glycerol nutrient broth (Oxoid) and stored at –80°C until needed. Prior to each experiment, bacterial cultures were refreshed from stocks on nutrient agar (Oxoid), and a fresh bacterial suspension was prepared for each of the strains grown overnight in 100 mL of nutrient broth (in 0.5 L Erlenmeyer flasks) at 37 °C with shaking (120 rpm). Bacterial cells were collected at the logarithmic stage of growth. To ensure that the samples possessed similar numbers of cells despite variations of cell densities among the different strains used, the cell density of each strain was adjusted to OD_600_ = 0.3.

Incubation of the bacterial cultures was carried out as follows. A 700 μL aliquot of bacterial suspension was placed on the substrate samples and allowed to incubate for 18 h at 25 °C. Five substrates of each surface type were used for each strain. Three independent experiments were performed to confirm the results. After incubation, the discs were gently washed with copious amounts of MilliQ water to remove any non-attached cells and allowed to dry at room temperature for 45 min at 55% humidity. Under these conditions, the bacterial cells were able to maintain a semi-hydrated state, which was confirmed by their morphological appearance. This allowed all imaging experiments to be performed under similar conditions.

Titanium discs containing attached bacteria were sputter-coated with gold using a Dynavac CS300 device using the protocol developed in our laboratory to allow the amount of attached bacteria to be determined using scanning electron microscopy,. High-resolution images were taken by FESEM (ZEISS SUPRA 40VP) at 3 kV under 1000, 5000 and 20000× magnification. Images with 1000 and 5000× magnification were used to estimate the number of bacteria attaching to the substrates.

In order to visualize viable bacteria and the extracellular polymeric substances (EPS) on the substrate surfaces, standard staining techniques were used. Thus, the bacteria were stained green with SYTO^®^ 9 green (Molecular Probes™, Invitrogen) and the non-viable cells was stained red with propidium iodide (Molecular Probes™, Invitrogen). Images of the bacteria attached to the substrate surfaces were recorded using a confocal scanning laser microscope (CSLM) Olympus Fluoview FV1000 Spectroscopic Confocal System. The system included an inverted microscope OLYMPUS IX81 (with 20×, 40× (oil), 100× (oil) UIS objective lenses) and was operated with multiple Ar, He and Ne laser lines (458, 488, 515, 543, 633 nm). The imaging software Fluoview FV 7.0 was employed to process the CSLM images. The number of attached cells counted on the SEM images (1000 × magnification) was transformed into a number of bacterial cells per unit area (10 images were taken per sample; three samples were analyzed per bacterium). The number of cells attached to the surfaces and the bactericidal efficiency are presented in Supplementary Tables S3 and S4. The numerical values in these tables were calculated as an average of 10 individual recordings. Cell densities were calculated from SEM images taken at a magnification of 3,000×. The number of viable and non-viable cells was determined processing the images using Gwyddion software^54^.

### Culturing and seeding the primary human fibroblast on Ti surfaces

The culturing of primary human fibroblasts (pHF) on Ti surfaces was performed according to the methods described in the approved Biosafety Project 2014/SBC01. The culture medium (Promocell) was supplemented with 2% fetal bovine serum (FBS), fibroblast growth factors (1 ng/mL) and insulin (5 μg/mL). Cells were cultured to 70–80% confluency and were then trypsinized using the Detach kit (Promocell). The AR-Ti and HTE-Ti samples were seeded with pHF at a density of 10,000 cells per cm^2^ for each independent experiment. After 1, 3 and 10 days incubation periods, the control and titanium surfaces were gently washed with PBS, fixed with 4% *p*-formaldehyde for 15 minutes, permeabilized in 0.1% Triton X for 5 minutes then blocked with 1% BSA for 60 minutes. Image-IT^®^ FX Signal Enhancer (Invitrogen) was also used during the fixation stage to enhance the fluorescent signals. Any fixed cells present on the substrate surfaces were treated with a primary anti-vinculin antibody (Sigma) overnight, followed by goat anti-mouse secondary antibody conjugated with Alexa Fluor 594 (Invitrogen). Actin filaments were visualized by staining the cells with Alexa Fluor 488 conjugated Phalloidin (Invitrogen). Cell nuclei were labeled using DAPI (Invitrogen). Samples with stained cells were then placed in a μ-Disc 35 mm culture disc (ibidi GmbH, Martinsried, Germany) for imaging in a Fluoview FV10i inverted microscope (Olympus, Tokyo, Japan). After incubation, all samples were washed with 10 mM PBS and fixed in 2.5% glutaraldehyde (Sigma) for 30 min, then gently washed with of 10 mM PBS and progressively dehydrated in graded ethanol solutions (30, 50, 70, 90 and 100% v/v). The ZEISS SUPRA 40VP field-emission scanning electron microscope (Carl Zeiss NTS GmbH, Oberkochen, BW, Germany), operated at 3 kV, was used to determine the attachment and proliferation of cells on the substrate surfaces.

## Additional Information

**How to cite this article**: Bhadra, C. M. *et al.* Antibacterial titanium nano-patterned arrays inspired by dragonfly wings. *Sci. Rep.* **5**, 16817; doi: 10.1038/srep16817 (2015).

## Supplementary Material

Supplementary Information
