# Supplementary material for: Antibacterial titanium nano-patterned arrays inspired by dragonfly wings
Source: Sci Rep. 2015 Nov 18;5:16817. doi: 10.1038/srep16817 (PMC4649496; doi:10.1038/srep16817)
Supplement: Supplementary Information [file srep16817-s1.pdf]

Supplementary information

Chris M. Bhadra[1], Vi Khanh Truong[1], Vy T. H. Pham[1], Mohammad Al Kobaisi[1], Gediminas Seniutinas[1], James Y. Wang[2], Saulius S. Juodkazis[1,3], Russell J. Crawford[1], Elena P. Ivanova[1]*

[1]School of Science and [2]School of Engineering, Faculty of Science, Engineering and Technology, Swinburne University of technology, PO Box 218, Hawthorn, Victoria, 3122 Australia

[3]Center for Nanotechnology, King Abdulaziz University, Jeddah 21589, Saudi Arabia

*Corresponding author: eivanova@swin.edu.au. Telephone: +61 (3) 9214 5137

S1. ImageJ analysis

SEM images were analysed using the ImageJ 1.48 software package with DiameterJ and OrientationJ plugins. Axial thinning transform and Dilate plots were produced using DiameterJ and showed the general pattern of the nano-wires from the top view perspective that is a nonspecific angular distribution. Due to the random nature of the nano-wires overlaying the surface DiameterJ did not produce an accurate estimate of the diameter of the nano-wires, hence the nano-wire diameter size distribution was statistically evaluated by 520 manual measurements to obtain an accurate estimate. This distribution was constructed using a 5 nm bracket, giving a peak height at 30 nm diameter, and an average peak height of 40.3 nm with a standard deviation of 20.0 nm. This wide distribution arises not only from the various diameters of the nano-wires, but rather the formation of the nano-wire bundles, counted as one in the manual measurements. Various orientation distributions of the top view SEM images were obtained at 70% minimum coherency and a 1 pixel Gaussian window σ. The colour surveys of these analyses are also produced using the OrientationJ plugin, all of which show a high degree of randomness in the nano-wire arrays.

S2. Mechanism of titanium dioxide formation

The hierarchical TiO_2 nano-wire arrays on the titanium substrates were synthesized via a single-step hydrothermal growth process: $K_2Ti_2O_4(OH)_2$ nano-wire arrays are prepared using Ti foil through an alkali hydrothermal process, then calcined at 400 °C to form $K_2Ti_6O_{13}$ and anatase TiO_2.

$$8TiO_2 + 2KOH \rightarrow K_2Ti_8O_{17} + H_2O$$

$$K_2Ti_8O_{17} \xrightarrow{400\ ^\circ C} K_2Ti_6O_{13} + 2TiO_2$$

S3. Characterisation of Ti nano-patterned array surfaces

Chemical elemental analysis of the AR-Ti and HTE-Ti samples was performed using XPS. As expected, titanium and oxygen were found in the greatest quantity on the top surfaces of both the AR-Ti and HTE-Ti surfaces. Trace amounts of calcium and carbon were also found. The presence of calcium could be due to the leaching of the glass vessel in which the Ti billets were immersed for the hydrothermal treatment. The presence of carbon as a source of

contaminant has been found to lower the water contact angle values of the AR-Ti surfaces. Table S2 represents the average number of retained cells for both *S. aureus* and *P. aeruginosa* on the AR-Ti and HTE-Ti surfaces, as analysed from SEM micrographs. It is quite evident from above table that *S. aureus* has a higher degree of preferential attachment than *P. aeruginosa* cells, however, in both cases it is evident that the cells are attaching in greater number to the smoother AR-Ti surface than that of the HTE-Ti surfaces[1]. The bacterial cells were found to be undamaged after attaching to the AR-Ti substrate, whereas the cells were found to be damaged on the HTE-Ti substrate surfaces..

The data presented in Table S3 shows the bactericidal efficiency of the AR-Ti and HTE-Ti surfaces towards the attachment patterns of *S. aureus* and *P. aeruginosa* quantified through confocal laser scanning microscopy. From the table, it can be seen that that the HTE-Ti were more efficient in inhibiting the growth of *P. aeruginosa* cells, with an almost 50% reduction in the cell viability on this substrate surface. This was not the case for the *S. aureus* cells, where only ~20% of the cells appeared to be damaged or fully destroyed by the action of the HTE-Ti surface. The bactericidal efficiency of the HTE-Ti surface could be attributed towards the presence of nano-wires on the top surface of the titanium samples. These nano-wires appear to have the ability of penetrating the cell membranes, thus resulting in the complete damaging of the cells[1].

Figure S1 represents the XPS spectra of the AR-Ti and HTE-Ti surfaces. The dominant doublet peaks found for the surfaces of the AR-Ti and HTE-Ti could be identified as Ti 2p3/2 (BE = 459.0 eV) and the Ti 2p1/2 (BE = 464.0 eV) peak could be attributed to the presence of TiO_2. The third, lowest energy peak is attributable to metallic Ti, with BE = 453 eV for Ti 2p3/2. The form in which oxygen occurs at the surface of titanium is also revealed in the O 1s peak (Figure 4), which shows three components: one associated with titanium oxide; another with $C{=}O/CO_3$ groups and the third with surface contamination with hydrocarbon (C-O).

The Raman spectra of the AR-Ti and HTE-Ti surfaces are presented in Figure S3. It can be seen that the AR-Ti surface mostly intensifies the major peaks for anatase titanium at 395.33, 273.83 and 121.95 cm^{-1}, respectively. The AR-Ti sample shows one major rutile peak at 578.99 cm^{-1}. The hydrothermally treated and the heat-treated titanium surfaces showed strong peaks at 126.74 and 386.06 cm^{-1}, respectively for the anatase phases. A weak peak representing rutile Ti was also observed at 278.56 cm^{-1}on the treated titanium surfaces. The

peaks of crystalline rutile were originally studied for an amorphous sample after strengthening at 400°C, with these peaks tending to become sharper as the annealing temperature is increased, indicating an increased crystallinity of the rutile phase.[2]. Additional strong peaks are also observed at 1563.95 and 1301.45 cm^{-1} in the spectra for the treated titanium surfaces. These peaks are highly indicative of the presence of amorphous carbon on all of the surfaces.

S4. Supporting tables and figures

Table S1. Elemental analysis of the AR-Ti and HTE-Ti surfaces.

Element	Peak	AR-Ti (A %)	HTE-Ti (A %)
Ti	2p	27.4	23.2
O	1s	66.8	64.2
C	1s	3.9	5.8
Ca	2p	1.6	3.0
K	2p	0.2	3.8

Table S2. Statistical topography and roughness distribution of the AR-Ti and HTE-Ti surfaces over a 46.7 × 62.3 μm^2 scan area.

	S_a (nm)	S_q (nm)	S_z (µm)	S_t (µm)
AR-Ti	356.93 ± 12.26	446.83 ± 16.95	3.46 ± 0.28	2.61 ± 0.29
HTE-Ti	401.35 ± 0.89	481.74 ± 0.05	3.56 ± 0.2	3.98 ± 0.21

[a]Roughness parameters are: average roughness (S_a), RMS roughness (S_q), average maximum profile (S_z) and maximum height (S_t).

Table S3. Attachment of bacterial cells on the surfaces of AR-Ti and HTE-Ti surfaces.

Substrate	$\times 10^4$ attached cells per mm^2	
	S. aureus	*P. aeruginosa*
AR-Ti	42.4 ± 4.9	6.3 ± 2.1
HTE-Ti	37.3 ± 17.2	5.2 ± 0.9

Table S4. Viability of bacteria upon attachment to the AR-Ti and HTE-Ti Surfaces.

	S. aureus ($\times 10^4$ attached cells per mm^2)			*P. aeruginosa* ($\times 10^4$ attached cells per mm^2)		
	Live	Dead	Viability (%)	Live	Dead	Viability (%)
AR-Ti	1.4 ± 0.24	0.1 ± 0.02	93.4	1.9 ± 0.04	0.4 ± 0.04	85.0
HTE-Ti	0.7 ± 0.03	0.18± 0.04	80.2	1.3 ± 0.25	1.2 ± 0.33	52.9

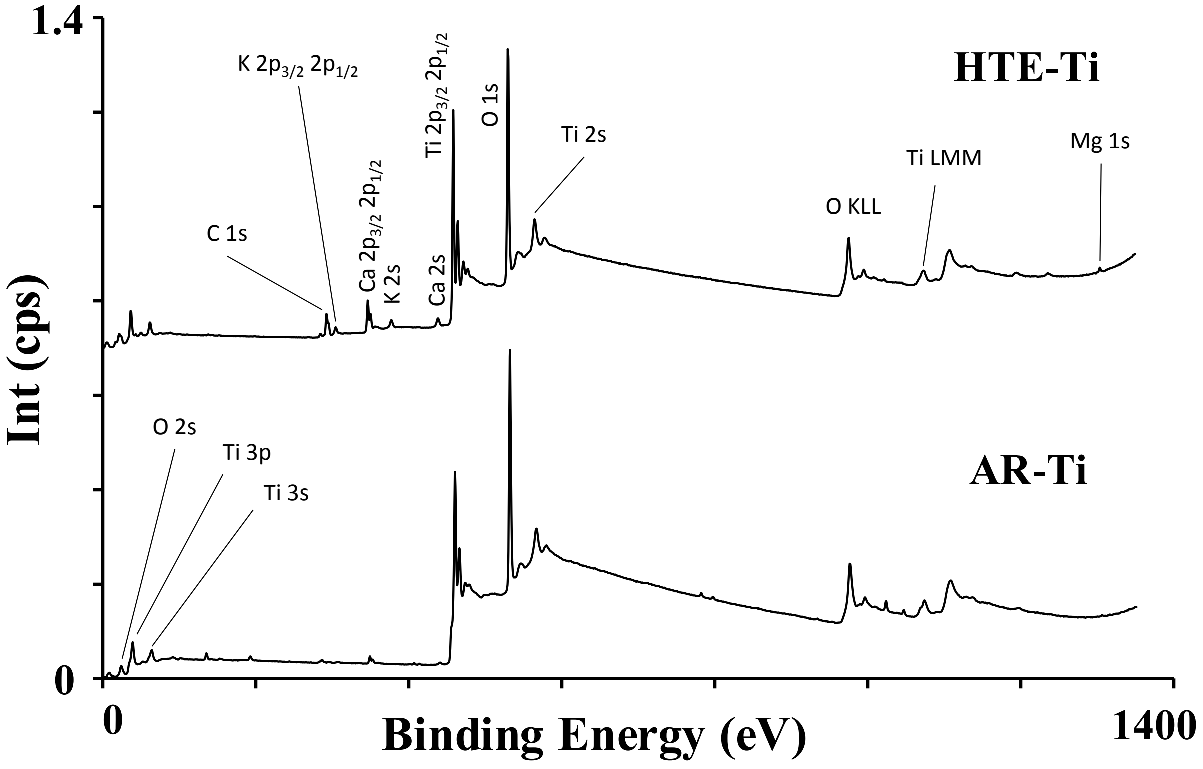

Figure S1. XPS-spectra of the AR-Ti and HTE-Ti surfaces.

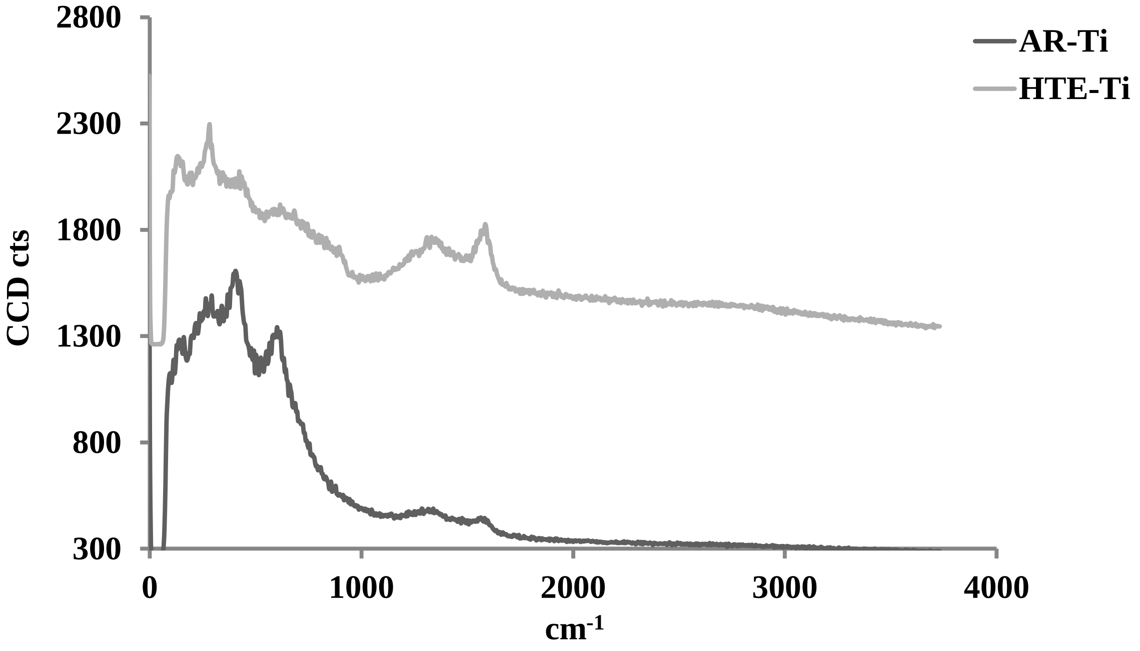

Figure S2. Raman-spectra of titanium surface topographies for the AR-Ti and HTE-Ti surfaces.

AR-Ti HTE-Ti

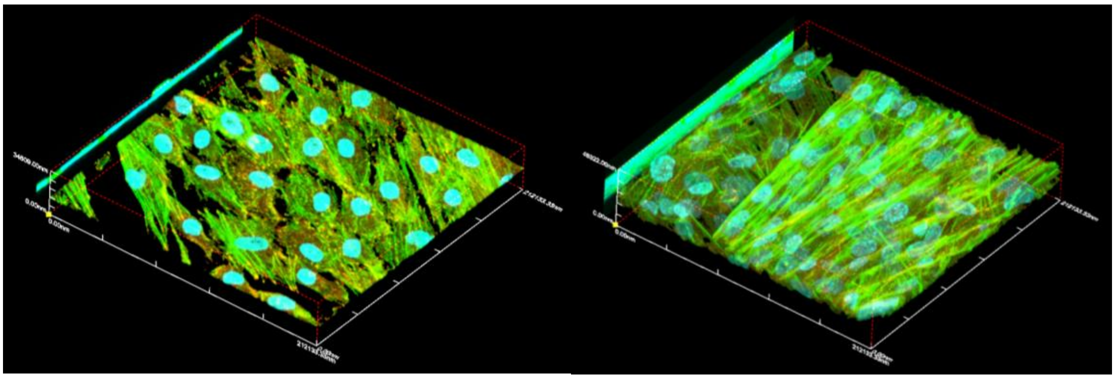

Figure S3. Fibroblast cell proliferation and differentiation on the AR-Ti and HTE-Ti surfaces after 10 days of incubation.

References

1. Truong, V.K., et al., *The influence of nano-scale surface roughness on bacterial adhesion to ultrafine-grained titanium*. Biomaterials, 2010. **31**(13): p. 3674-3683.
2. Hardcastle, F., *Raman spectroscopy of titania (TiO 2) nanotubular water-splitting catalysts*. Journal of the Arkansas Academy of Science, 2011. **65**: p. 43.
